# Effects of Botulinum Toxin Type A on Collagen Deposition in Hypertrophic Scars 

**DOI:** 10.3390/molecules17022169

**Published:** 2012-02-21

**Authors:** Zhibo Xiao, Guofan Qu

**Affiliations:** 1 Department of Plastic and Aesthetic Surgery, The Second Affiliated Hospital of Harbin Medical University, Harbin 150086, China; 2 Department of Orthopaedics, The Third Affiliated Hospital of Harbin Medical University, Harbin 150086, China

**Keywords:** botulinum toxin type A, collagen, hypertrophic scar

## Abstract

A recent study reported that Botulinum toxin type A (BTXA) could inhibit the growth of hypertrophic scars and improve their appearance. However, the mechanism of BTXA’s action on hypertrophic scars is still unknown. Some *in vitro* studies had shown BTXA could alleviate hypertrophic scars by acting on the biological behavior of fibroblasts, but there are few *in vivo* experiments, especially animal model experiments, supporting these findings. The aim of the study reported herein was to investigate the effect of BTXA on collagen deposition on hypertrophic scars in a rabbit ear model and partially clarify the mechanism of BTXA on the hypertrophy of scars. The rabbit hypertrophic scar model was used and eight rabbits were employed. BTXA was injected into the hypertrophic scar tissue of one ear; and the other ear in the same rabbit was the control without BTXA injection. The scar thickness and deposition of collagen was examined through immune histochemistry including haematoxylin and eosin (H&E) and Masson trichrome staining. The thicknesses of hypertrophic scars in the BTXA treatment group were obviously lower than in the control groups (*P* < 0.01). H&E and Masson staining showed that collagen fibers were stained blue. Compared with the treatment group, the collagen fibers were thicker and the arrangement of collagen fibers were disordered in the control group. This study used the rabbit ear model of hypertrophic scars to assess the effects of BTXA on scar hypertrophy. The application of BTXA may be useful for inhibiting hypertrophic scars.

## 1. Introduction

Hypertrophic scars, which are cosmetically and functionally unappealing, are characterized by excessive fibrosis and extra cellular matrix (ECM) deposition. Due to the fact that the etiology of hypertrophic scar formation has not been fully delineated, clinical management remains problematic [1]. Numerous treatments are currently available, including surgical excision, steroid injection, radiation therapy, laser and pressure therapy, but these methods cannot always provide good therapeutic results. Hence, alternatives are needed.

Some scholars found that BTXA could inhibit the growth of hypertrophic scars and improve the appearance of hypertrophic scars in clinical experiments [2]. These clinical observations were reported several years ago [3]. In order to elucidate the mechanisms of BTXA’S action on hypertrophic scars, some *in vitro* studies had been carried out. The authors reviewed some previously published articles regarding this problem and summarized some of the latest advancements [2,3]. Firstly, some scholars had reported that BTXA could promote the atrophy of benign prostatic hyperplasia by inducing apoptosis of prostatic epithelium and inhibiting the growth of prostate cancer by inducing apoptotic cancer cells. BTXA could also induce temporary apoptosis of nasal glandular cells. These articles led the authors to consider the relationship between BTXA and cellular dynamics of fibroblasts derived from hypertrophic scars. The authors had already carried out some experimental research and found that BTXA could influence the cell cycle of fibroblasts derived from hypertrophic scars, inhibiting the proliferation and promoting apoptosis of fibroblasts [4,5,6]. Secondly, BTXA could be closely associated with transforming growth factor (TGF-β1). As is known, TGF-β1 plays an important role in the formation of hypertrophic scars. The high expression of TGF-β1 has obviously promoted the formation and growth of hypertrophic scars. Recent reports have shown that BTXA could reduce the expression of TGF-β1 protein in fibroblasts derived from *in vitro* experiments [7,8,9]. The latest advancement mentioned may partially explain the molecular mechanism of action of BTXA on hypertrophic scars. Although these findings deepened our understanding regarding the mechanism of BTXA’s action on hypertrophic scars, the authors still believed the latest advancement was not enough to elucidate the biological mechanism. Limitations of the above findings cannot be ignored due to these finding being obtained only in fibroblasts cultured *in vitro*. The *in vitro* environment may cause some limitations of the experimental results. In the other word, almost no *in vivo* studies regarding the problem have been reported, and no animal experiments have been carried out to strengthen these findings. Thus, it is necessary to perform animal experiments for strengthening the findings. The study will offer the *in vivo* experimental results which help to understand the mechanism of BTXA’s on hypertrophic scars. The aim of this study was to investigate the effects of BTXA on collagen deposition of hypertrophic scars in the rabbit ear model.

## 2. Results and Discussion

### 2.1. Results

**Figure 1:**
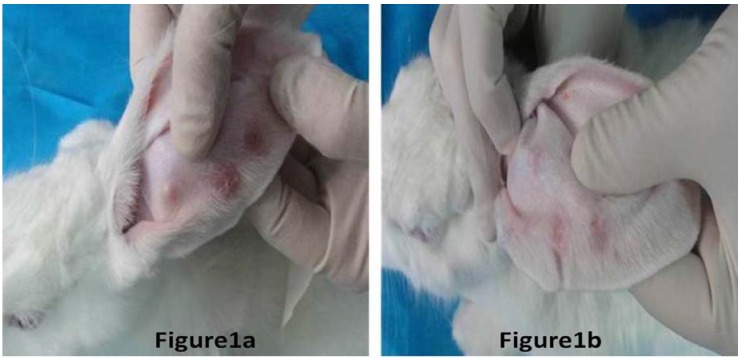
Hypertrophic scar in the rabbit ear. Figure 1a represents the hypertrophic scar without BTXA treatment, and the scar was followed up after six months of surgical trauma in the rabbit ear model. Figure 1b represents the hypertrophic scar with BTXA treatment, and the scar was followed up after six months of surgical trauma in the rabbit ear model. Comparing the two figures, they showed that BTXA could improve the symptoms of hypertrophic scars.

**Figure 2:**
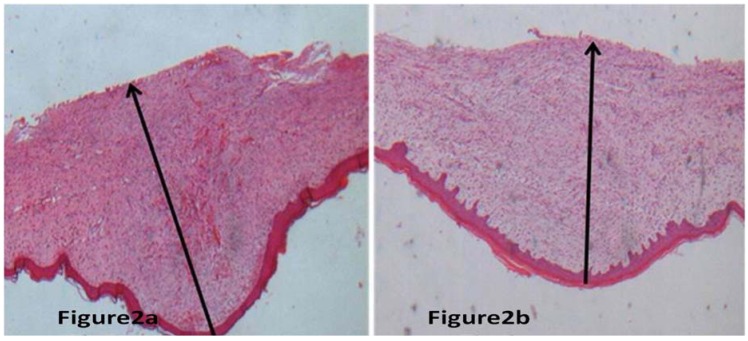
Represented hypertrophic scar stained by H&E stain. Measurement of the thickness of scar at 100 magnification. Figure 2a,b represented scar without BTXA treatment and the scar received BTXA treatment, respectively. Through the microscopic observation, it was found that the thickness of scar received BTXA treatment was thinner than that of scar without BTXA treatment.

The thickness of scars with BTXA treatment decreased significantly in comparison to the non-treated control scars (*P* < 0.01); for more details see Table 1. We could find that the scar thickness decreased significantly in hypertrophic scars with BTXA treatment in comparison to their non-treated control scars by macroscopic and microscopic observation (Figure 1 and Figure 2). 

**Figure 3:**
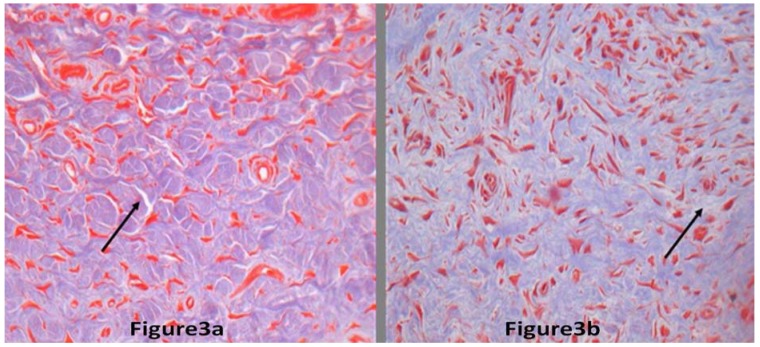
The collagen fibers of hypertrophic scars. The black arrow indicates collagen fiber (200× magnification). Figure 3a,b represents scars with BTXA treatment and the scar without BTXA treatment, respectively. The collagen fibers without BTXA treatment were thicker than those with BTXA treatment. In addition, the arrangement of collagen fiber without BTXA treatment was more disordered than those with BTXA treatment (Masson stain).

H&E and Masson staining showed that collagen fibers were stained blue. Collagen fiber was thinner markedly in the BTXA treatment group than in the control group. Collagen fibers were disorderly arranged and stained deeply in control group, but they were orderly arranged and stained slightly in the BTXA treatment group (Figure 3).

**Table 1:** Comparision of the thickness of two groups of hypertrophic scars.

**Group difference**	No of rabbits							
							
**Scar thickness** **Without BTXA (mm) **	1.92 ± 0.05	1.74 ± 0.03	1.57 ± 0.09	1.68 ± 0.02	1.37 ± 0.11	1.72 ± 0.8	1.64 ± 0.12	1.83 ± 0.09
**Scar thickness** **With BTXA (mm)**	0.90 ± 0.03	0.95 ± 0.18	1.16 ± 0.06	1.03 ± 0.11	1.08 ± 0.05	0.72 ± 0.09	0.69 ± 0.07	0.94 ± 0.02

### 2.2. Discussion

Hypertrophic scars are a benign hyperproliferative growth of dermal collagen. The patients suffering from hypertrophic scars often experience major physical (deformities, restricted range of motion, pain, and pruritus) and psychological (cosmetic concern) problems. Because the etiology for hypertrophic scar formation has not been fully delineated, clinical management remains problematic. Numerous treatments are currently available, including surgical excision, steroid injection, radiation therapy, laser, and pressure therapy, but these methods cannot always provide good therapeutic results sometimes [10]. Hence, it is necessary to explore new therapeutical methods for hypertrophic scars.

Previous reports had shown that BTXA could improve the appearance of hypertrophic scars and inhibit the growth of hypertrophic scars. In the initial stages of our understanding about the mechanism of BTXA’s action on hypertrophic scars, we believed that BTXA could be used in controlling hypertrophic scars due to the temporary denervation of BTXA. As we all know, tension is one of the chief factors determining the degree of scar formation. To explain the reason behind BTXA’s effects on bundles of collagen fibers, we reviewed some previously published literatures [11,12]. BTXA blocks the release of Ach at neuromuscular junctions and therefore primarily affects muscle tension. There are the thinnest muscle fibers under subcutaneous tissue of rabbit ear. Due to the decreasing tension in close proximity to the scar, local fibroblasts gradually changed their functional status, causing them to proliferate slowly and synthesize less extracellular matrix, including collagen. Moreover, fibroblasts in the condition of weak tension, caused by BTXA, secreted less of some biologically active mediators, thus inhibiting fibroblasts to proliferate drastically and synthesize much extracellular matrix. These reasons resulted in improved hypertrophic scars [13]. However, with the fast advances of academic research on the mechanism of BTXA’s action on hypertrophic scars, the molecular nets of the biological mechanism between BTXA and hypertrophic scars have been further elucidated. Previous studies have demonstrated that BTXA could promote the apoptosis and inhibit the proliferation of fibroblasts derived from hypertrophic scars in an *in vitro* study [14]. Additionally, BTXA could inhibit the expression of TGF-β1 protein in hypertrophic scar fibroblasts in *in vitro* experiments [15]. These latest advancements denoted that the mechanism of BTXA’s action on hypertrophic scars was very complicated, which let us know what we had learned about the mechanism was insignificant and the molecular mechanism remains unknown. 

Most of recent studies paid more attention to the effect of BTXA on fibroblasts derived from hypertrophic scars. Many scholars have emphasized the regulation of BTXA on the apoptosis and proliferation of fibroblasts, and most advancements about this problem emerged on the basis of *in vitro* experiments and clinical observations. Few researchers have focused on BTXA’s influence on collagen deposition in hypertrophic scars. Especially, we could barely find *in vivo* experiments on these problems. The authors believed the deposition of collagen was closely associated with the degree of hypertrophy of scars. Therefore, it was necessary to explore BTXA’s action on collagen deposition in an animal model.

In the study, the authors chose the rabbit ear model of hypertrophic scars. Although some animal hypertrophic scar models were used, an ideal animal model of hypertrophic scarring is unavailable. Any one type of animal models of hypertrophic scar has both its advantages and disadvantages. All the hypertrophic scar models were divided into two categories. One is the nude mice animal model in which human scars have been transplanted. Although the model of nude mice has some advantages, there exist vital weaknesses in the nude mice model. As we all know, wound healing is based on immunity. If immunity is lacking, wound healing will be severely influenced. Hypertrophic scars are based on the result of wound healing. Thus, immunity exerts a considerable effect on the formation of hypertrophic scars. Nude mice lack immunity, which leads to the fact that nude mice animals have a vital weakness in the study of hypertrophic scars. Apart from the nude mice model, rabbit ears were used in producing the hypertrophic scar model. The model of rabbit ear does well in reflecting the stages of scar development. The course of hypertrophy of the scar in rabbit ear is nearly similar to the course in human beings. In addition, the rabbit ear model maintains good immunity. Thus, the authors chose the rabbit ear model instead of the nude mice model 

Through observation of the rabbit ear model, the authors found that BTXA could inhibit the hypertrophy of scars in the rabbit ear model, which provided the *in vivo* experimental evidencs that BTXA could inhibit hypertrophic scars. Most importantly, the study was the first report that proved the effects of BTXA on collagen deposition of hypertrophic scars. The data in the study indicated that BTXA treatment and control groups displayed distinctive collagen deposition and the degree of hypertrophy of scars, which partly explain why BTXA could improve the eventual appearance of hypertrophic scars and inhibit their growth in clinical treatment. The content of this paper represents the preliminary results of our study, and it does not elucidate the reasons why BTXA affected the collagen deposition of hypertrophic scars. The authors believe that the paper had some shortcomings because it didn't elucidate the molecular mechanism of BTXA’s action on the degree of hypertrophy of scar. These limitations couldn’t be ignored. Thus, the evaluation of collagen secretion ability of fibroblast or examination of mRNA expression of collagen fiber directly would strengthen our findings. Our next study will focus on the *in situ* hybridization and reverse transcriptase polymerase chain reaction analysis of the original specimens. We have noticed that TGF-β1 is the key cytokine in formation of hypertrophic scars. TGF-β1 not only regulates cellular growth, differentiation, adhesion, and apoptosis, it also causes excessive deposition of collagen. Thus, TGF-β1 has been regarded as an important cytokine closely related to the formation and growth of hypertrophic scars. In *in vitro* experiments, we had already elucidated that BTXA caused a decrease in TGF-β1 protein. These findings led to the hypothesis that the effects of BTXA on the collagen of hypertrophic scars may be through its reduction of TGF-β1 secretion in *in vivo* experiments. This will become the investigative emphasis of our next work. In summary, this article is the first time, to our knowledge, that the collagen deposition of hypertrophic scar is reported to have significantly decreased through the application of BTXA in an *in vivo* experiment. 

## 3. Experimental

### 3.1. Hypertrophic Scar Model

Eight young adult New Zealand White rabbits weighing between 2.5 and 3.5 kg were used in this study. The animals were handled according to procedures approved by the Harbin Medical University Animal Care and Use Committee. After the animals were anaesthetized with ketamine (60 mg/kg) and xylazine (5 mg/kg), the authors followed the protocol for initiating the hypertrophic scars as previously described [16]. Briefly, three wounds were created down to bare cartilage on the ventral surface of each ear by means of a 8-mm punch biopsy at standardized locations. A dissecting microscope was used to ensure removal of the epidermis, dermis and perichondriumin each wound. Removal of the perichondrial layer delayed epithelialisation of the 8-mm defect, which supports hypertrophic scar formation. Haemostasis was then obtained by applying pressure and each wound was individually covered with Tegaderm dressing until the entire wound appeared re-epithelialized.

### 3.2. Scar Treatment and Measurement

For treatment the rabbits had three wounds on each ear. The scar on the right ear of every rabbit was treated with BTXA; and the lesion on the left ear of every rabbit wasn’t injected with BTXA as control. On the postoperative 28 days, the rabbits were sedated. BTXA (produced by Lanzhou Biochemical Company, Lanzhou, China) was applied. All the rabbits were treated once a month with intralesional BTXA for a total of three months. The solutions were injected into the body of the lesion using a gauge needle until slight blanching was visible. The BTXA dosage was adjusted to 0.5 U per cubic centimeter of lesion, but did not exceed 5 U per rabbit in one injection. The rabbits were sacrificed after three months of the last BTXA injection and the scars were harvested. The scars were bisected through the point of maximum height of hypertrophic scare. Each scar was fixed in 4% neutral buffered formaldehyde, dehydrated, embedded in paraffin, cut in 4-mm sections, and stained with haematoxylin and eosin (H&E) and Masson trichrome. Scar thickness was quantified. The thickness of hypertrophy of each scar was expressed as data, whose measurements were based on those made from haematoxylin eosin-stained tissue sections at 100× magnification. The degree of magnification for the Masson trichrome-stained tissue was 200× magnification.

### 3.3. Statistical Analysis

Each sample was evaluated by a blinded examiner using a calibrated eyepiece reticule at different time points. The values were averaged. Comparisons were made between the treatment groups and their control groups using the Student’s t-test. The level of significance was set to *P* values less than 0.05. Data are presented as mean values ±SD of the scar thickness.

## 4. Conclusions

The current studies provide evidences that BTXA can inhibit hypertrophic scars in clinical work and *in vitro* experiments. No animal experiments about BTXA’s influence on collagen deposition has been reported. This study used the model of hypertrophic scar on the rabbit ear to assess the effects of BTXA on the hypertrophy of scars in *in vivo* experiments. The results represented here suggest that using BTXA to inhibit hypertrophy scars was effective in the rabbit model and the application of BTXA may be useful for inhibiting hypertrophic scars.
